# Autophagy Is Possibly Involved in Osteoblast Responses to Mechanical Loadings

**DOI:** 10.3390/cimb44080247

**Published:** 2022-08-11

**Authors:** Yanghui Xing, Liang Song, Yingying Zhang

**Affiliations:** 1 Department of Biomedical Engineering, Shantou University, Shantou 515063, China; 2 Beijing Key Laboratory of Rehabilitation Technical Aids for Old-Age Disability, National Research Center for Rehabilitation Technical Aids, Beijing 100176, China

**Keywords:** osteoblast, mechanotransduction, autophagy, ATP

## Abstract

Both mechanical loading and autophagy play important roles in regulating bone growth and remodeling, but the relationship between the two remains unclear. In this study, we examined bone structure with micro-CT imaging and measured bone mechanical properties with three-point bending experiments using bones from wild-type (WT) mice and conditional knockout (cKO) mice with Atg7 deletion in their osteoblasts. We found that the knockout mice had significantly less bone volume, bone thickness, bone ultimate breaking force, and bone stiffness compared to wild-type mice. Additionally, bone marrow cells from knockout mice had reduced differentiation and mineralization capacities in terms of alkaline phosphatase and calcium secretion, as well as Runx2 and osteopontin expression. Knockout mice also had significantly less relative bone formation rate due to mechanical loading. Furthermore, we found that the osteoblasts from wild-type mice had stronger responses to mechanical stimulation compared to autophagy-deficient osteoblasts from knockout mice. When inhibiting autophagy with 3 MA in wild-type osteoblasts, we found similar results as we did in autophagy-deficient osteoblasts. We also found that mechanical loading-induced ATP release is able to regulate ERK1/2, Runx2, alkaline phosphatase, and osteopontin activities. These results suggest that the ATP pathway may play an important role in the possible involvement of autophagy in osteoblast mechanobiology.

## 1. Introduction

It is well known that mechanical loading of bone results in a variety of biophysical signals that affect bone cell growth and differentiation. Among all bone cells, osteoblasts are the bone-forming cells and are responsible for bone growth and remodeling. Osteoblasts are sensitive to mechanical stimulation, which can activate a number of intracellular signaling pathways regulating osteoblast activities [1,2,3]. Autophagy is a natural catabolic process that uses lysosomes to degrade unwanted or damaged cellular components in an orderly manner. It is essential for cell growth, survival, differentiation, development, and homeostasis [4,5]. Autophagy deficiency has been implicated in a number of bone diseases, such as osteoporosis, Paget’s disease of bone, and osteopetrosis [5,6,7]. Previous literature showed that inhibiting autophagy in mice leads to osteoporosis, which resembles skeletal aging [8], and autophagy deficiency in osteoblasts also inhibits terminal differentiation of these cells [9]. Moreover, autophagy is related to osteoblast mineralization, possibly because autophagic vacuoles can be used to transport apatite crystals to the extracellular space [10], and defective autophagy in osteoblasts induces endoplasmic reticulum stress and causes remarkable bone loss [11]. However, the deletion of autophagy receptor NBR1 may negatively affect osteoblastic bone formation via the p38 MAPK pathway [12]. Therefore, the role of autophagy in bone biology requires further investigation.

Recently, the relationship between mechanical stimulation and autophagy has received significant attention. Mechanically induced autophagy has been shown to be associated with ATP metabolism and cellular viability in osteocytes in vitro [13]. Another study found that the chaperones Hsc70 and HspB8 in smooth muscle cells form a complex to sense tension and subsequently trigger autophagy mechanisms to remove damaged filamin [14]. King et al. showed that mechanical compression significantly enhanced the expression of the autophagy marker protein LC3-II in MDA-MB-231 cells [15,16]. Moreover, shear stress induced by blood flow activates cell autophagy pathways in vascular endothelial cells and subsequently regulates eNOS and ET-1 production [17,18]. These studies have suggested that mechanical stimuli are able to activate autophagy pathways in various cell types. On the other hand, osteocytes can promote osteoclastogenesis via autophagy-mediated RANKL secretion under mechanical compressive force [19]. Previous literature has demonstrated that autophagy-deficient mice have reduced bone volume [9,10], but how autophagy affects the ability of osteoblasts to sense mechanical loading is largely unknown.

In response to mechanical loading, osteoblasts release ATP, which subsequently initiates ERK1/2 activation, PGE2 release, and intracellular calcium pathways [20,21]. ATP is also able to stimulate differentiation and mineralization of osteoblastic cells [10,22]. Additionally, previous studies have shown that ATP may be released from cells via autophagic vesicles [23,24]. Thus, in this study, our aim was to investigate the role of ATP in autophagy-induced osteoblast responses under mechanical loading.

## 2. Materials and Methods

### 2.1. Animal and Cell Culture

Conditional Atg7 knockout mice were generated by crossing Atg7-floxed mice with mice expressing Cre recombinase under the control of the osteocalcin promotor (Casgene Biotechnology Inc., Beijing, China) [25]. Briefly, mice expressing Cre recombinase under the control of the osteocalcin promoter (abbreviated as OC-Cre/Atg7^+/+^) were bred with mice in which the Atg7 gene is flanked by two loxP sites (Atg7^flox/flox^, acquired from RIKEN Bio Resource Center) [26] to generate OC-Cre/Atg7^flox/+^ mice. Next, we crossed OC-Cre/Atg7^flox/+^ with Atg7^flox/flox^ to generate OC-Cre/Atg7^flox/flox^. Next, we back-bred OC-Cre/Atg7^flox/flox^ mice with Atg7^flox/flox^ mice to generate an equal number of OC-Cre/Atg7^flox/flox^ (Atg7 deficient) and Atg7^flox/flox^ (wild type) littermate mice. PCR analysis confirmed deletion of the floxed sequence in the genomic DNA from these mice. Only male mice were used for bone structure phenotype studies to eliminate the effects of female hormones on bone characteristics.

Mice at age of 8 to 12 weeks were sacrificed and their bone marrow cells and osteoblasts were extracted according to our previous methods [21]. Briefly, marrow cells were flushed out from femurs and tibias with syringes, and osteoblasts were collected from bone chip cultures. Bone marrow cells were subcultured in 12-well plates for differentiation and mineralization with cell differentiation medium (α–MEM with 10% FBS, 50 mg/mL L-Ascorbic Acid, 10 nM Dexamethasone, 10 mM Beta-Glycerol-phosphate, 1% P/S). For drug treatment of cells, the concentration of 3 MA is 5 mM, and the concentration of apyrase is 10 U/mL.

### 2.2. Analysis of Bone Structure and Mechanical Properties

Femurs from the right side of 16-week-old mice were harvested for micro-CT analysis. Periosteal volume, endosteal volume, bone porosity, bone thickness, and BMD were calculated for the diaphysis of each femur using the Scanco Image Processing Library routines. For measurements of bone mechanical properties, femurs from 16-week-old mice were stored in PBS before being mechanically tested to failure in three point bending experiments using a TA Electroforce 3220 testing machine. All testing was executed with bones that were hydrated and at ambient temperature. Protocol details were described in our earlier studies [21].

### 2.3. Alkaline Phosphatase (ALP) and Calcium Analysis

For ALP staining, a commercially available kit (Sigma, St. Louis, MO, USA) was used. Cells were fixed and stained following the manufacturer’s instructions. For quantification, AP activity was determined by the colorimetric conversion of p-nitrophenol phosphate to p-nitrophenol (Sigma, St. Louis, MO, USA) and normalized to total protein (Pierce). Extracellular calcium was identified using the von Kossa method. Cells were fixed with 4% formaldehyde, then incubated with 5% sliver nitrate for 20 min, and rinsed with distilled water three times. To quantify calcium, the o-cresolphthalein method was used following instructions from the calcium assay kit (Cayman Chemicals, Ann Arbor, MI, USA) [21].

### 2.4. Western Blot

Western blot experiments were carried out using existing protocols. Briefly, cytosol proteins were extracted with cell lysis buffer, then protein concentrations were measured using a protein assay kit from Pierce. Normalized amounts of crude proteins were next electrophoresed in SDS-PAGE gel, and then PVDF membranes were used for protein transfer. β-actin served as a housekeeping protein control. Quantification of Western blots was carried out using a Bio-Rad GS-800 densitometer and Quantity One image analysis software.

### 2.5. Bone Formation Rate Analysis

Mice at 16 weeks were anesthetized with isofluorane and anesthesia maintained with isofluorane throughout the entire loading regime. The right ulnas of mice were placed in the loading device and subjected to a compression of 2.1 N which induced 2500 μ strain at the ulna midshaft based on strain gauge measurement and numerical analysis. Micrometer heads and a series of slots designed into the in vivo loading platform allowed for reproducible placement of each animal over the successive loading bouts. The ulna was loaded with 120 cycles at 2 Hz for 3 consecutive days. Mice received calcein and alizarin labeling on day 5 and day 11. Mice were then euthanized on day 17. The ulnas were then removed for histomorphometry to determine the bone formation rate as described previously [27].

### 2.6. Oscillatory Fluid Flow Stimulation

Oscillatory fluid flow stimulation was used as the mechanical loading for cells. Osteoblasts were subcultured onto glass slides and subjected to oscillatory fluid flow at 10 dynes/cm^2^ and 1 Hz as described previously [28]. Based on our previous experiments, cells were subjected to 1 min or 15 min of oscillatory fluid flow in ATP release studies, 5 min or 15 min in ERK1/2 activation studies, and 60 min in Runx2 activation studies, 60 min per day for three days in mRNA studies.

### 2.7. Quantitative PCR Analysis

Cells were lysed and homogenized with a QIAshredder mini column (QIAGEN, Germantown, TN, USA). Total RNA was extracted with Qiagen RNeasy mini kit. cDNA was prepared from 1 mg total RNA using the iScript Kit (Bio-rad, Hercules, CA, USA). RT-qPCR was performed with Thermo StepOne Plus Real-Time PCR System, and data were analyzed with the DataAssist^TM^ program. The primers were Runx2 forward: 5′-ACG AGG CAA GAG TTT CAC CTT GAC-3′, Runx2 reverse: 5′- AGG TAG CTA CTT GGG GAG GAT TTG-3′; ALP forward:5′- TGC GCA GGA TTG GAA CAT CAGT-3′, ALP reverse: 5′- TGC ACC CCA AGA CCT GCT TTAT-3′; osteopontin (OPN) forward 5′-TAC GAC CAT GAG ATT GGC AGT GA-3′, OPN reverse: 5′-TAT AGG ATC TGG GTG CAG GCT GTAA-3′. The primers for mouse β-actin as controls were forward 5‘-AGA GGG AAA TCG TGC GTG AC-3′ and reverse 5‘-CAA GAA GGA AGG CTG GAA AA-3′.

### 2.8. Statistics

Experimental results were tested using the statistical software MINITAB (V11, Mintab, LLC, State College, PA, USA). Data are shown as the mean ± SEM. A two-sample Student’s *t*-test was used to examine the difference between groups. A star sign (*) on a bar graph indicates when the *p* value was smaller than 0.05, which implies significant statistical difference.

## 3. Results

### 3.1. Autophagy Deficiency in Osteoblasts Decreases Bone Mass and Mechanical Properties

**Figure 1:**
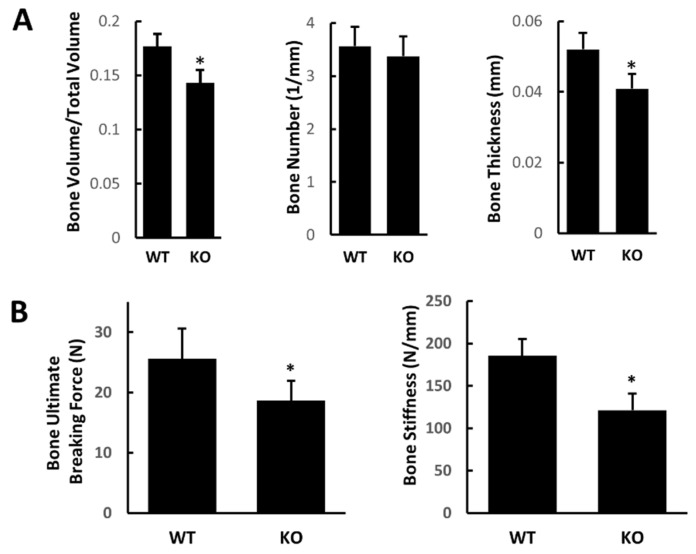
Autophagy-deficient mice have reduced bone mass and bone mechanical properties. (**A**) micro-CT analysis of bone structure within WT and Atg7 cKO mice. Trabecular bone volume, bone number, and bone thickness were quantified. (**B**) Three-point bending test of femurs to examine bone ultimate breaking force and bone stiffness. (*n* = 6, * *p* < 0.05. Error bar represents the mean ± SEM).

With micro-CT imaging, we analyzed the femoral structure of WT and Atg7 cKO mice at 16 weeks of age and found that the trabecular bone volume and thickness were significantly decreased in Atg7 cKO mice relative to WT mice (Figure 1A). We then examined the bone mechanical properties of WT and Atg7 cKO mouse femurs at 16 weeks. We found that Atg7 cKO mice also had significantly lower bone ultimate breaking force and bone stiffness compared to WT mice (Figure 1B). These results suggested that Atg7 may be involved in bone growth and remodeling.

### 3.2. Autophagy Deficiency Decreases Osteoblast Differentiation and Mineralization Capacities

**Figure 2:**
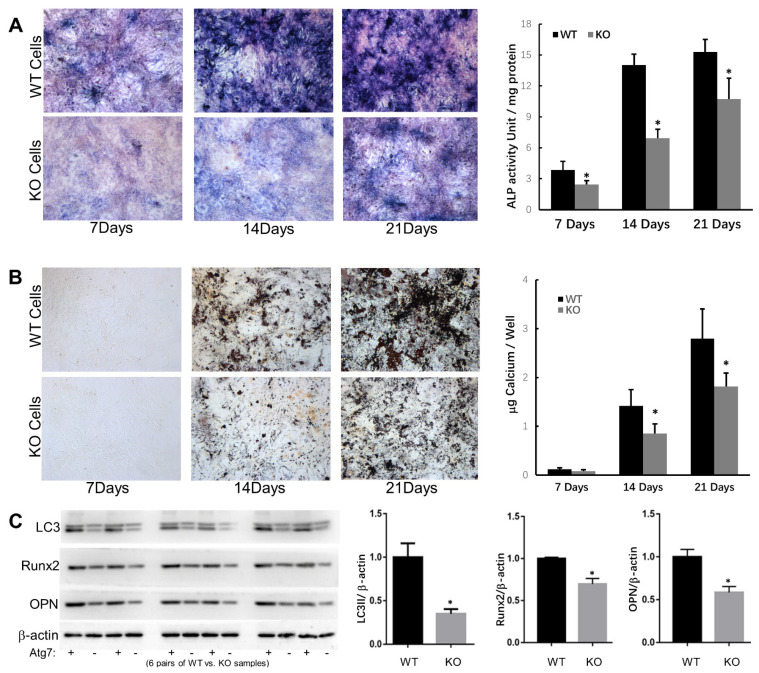
Differentiation and mineralization capacities were decreased in Atg7 KO osteoblasts. (**A**) Images of ALP staining and quantification of bone marrow cell cultures. (**B**) Images of von Kossa staining and qualification of bone marrow cell cultures. (**C**) Protein expression of LC3, Runx2, and osteopontin in WT and Atg7 KO osteoblasts. (*n* = 6, * *p* < 0.05. Error bars represent SEM).

Bone marrow cells were cultured in 12-well plates for 7, 14, and 21 days in cell differentiation media and then stained for ALP and calcium. We found that the ALP activity in WT cells was significantly higher than that in Atg7 KO cells at days 7, 14, and 21 (Figure 2A). Similarly, WT cells had significantly more calcium deposition than Atg7 KO cells (Figure 2B). Additionally, we examined the protein expression of Runx2 and osteopontin (OPN), both of which are important to osteoblast proliferation and differentiation, and we found that their expressions were higher in WT cells (Figure 2C). These results may partially explain the osteopenic phenotype of Atg7 cKO mice.

### 3.3. Autophagy Deficiency in Osteoblasts Decreases Mechanical Loading-Induced Bone Formation Rates

**Figure 3:**
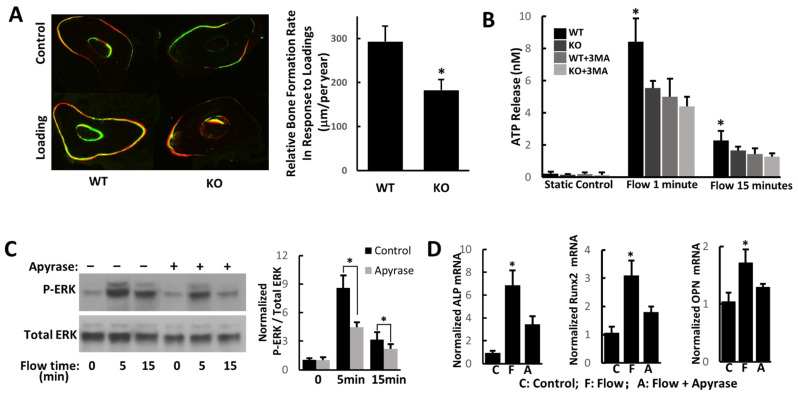
Autophagy is involved in osteoblast mechanotransduction. (**A**) Relative bone formation rates at the ulnar midshaft (loaded side minus unloaded side), which were induced by external mechanical loading, were significantly decreased in autophagy-deficient mice compared to wild-type mice. (**B**) ATP release of WT and Atg7 KO osteoblasts in response to oscillatory fluid flow with and without 3 MA. (**C**) ERK1/2 phosphorylation in response to oscillatory fluid flow with and without apyrase. (**D**) Osteoblast gene expression of ALP, Runx2, and osteopontin with and without apyrase. (*n* = 5, * *p* < 0.05; each bar represents the mean ± SEM).

The bone formation rate is an important parameter in bone remodeling. Using the left ulna (non-loaded) values subtracted from the right ulna (loaded) values, the relative bone formation rate represents a purely mechanically induced bone formation. Our results demonstrated that Atg7 cKO mice had a more than 30% reduction in the bone formation rate in response to ulnar compression compared to wild-type mice (Figure 3A), suggesting that the autophagy process may be involved in bone mechanotransduction.

### 3.4. Autophagy Affects ATP Release from Osteoblasts in Response to Mechanical Loading

We next examined ATP release from osteoblasts from both wild-type and Atg7 cKO mice. We found that the amount of ATP released by wild-type cells after oscillatory fluid flow was significantly higher than that in Atg7 KO cells. When we treated cells with autophagy inhibitor 3 MA, we found that the amount of ATP released from wild-type cells was significantly decreased in response to mechanical stimulation, while the amount of ATP released from Atg7 KO cells showed no significant changes after 3 MA treatment (Figure 3B).

### 3.5. ATP Modulates Osteoblast ERK1/2, Runx2, ALP, and OPN Activities in Response to Mechanical Loading

As mechanical stimulation can cause ATP release, we then examined the effects of oscillatory fluid flow on osteoblasts with or without apyrase, which is an enzyme able to hydrolyze ATP quickly and thus eliminate the effects from ATP release. We found that oscillatory fluid flow enhanced ERK1/2 phosphorylation and ALP, Runx2, and osteopontin gene expression in osteoblasts. However, the addition of apyrase significantly attenuated these effects (Figure 3C,D). ERK1/2, ALP, Runx2, and osteopontin have been shown to all be closely related to osteoblast proliferation and bone matrix production.

### 3.6. Autophagy Affects Osteoblasts ERK1/2, Runx2, ALP, and OPN Activities in Response to Mechanical Loading

**Figure 4:**
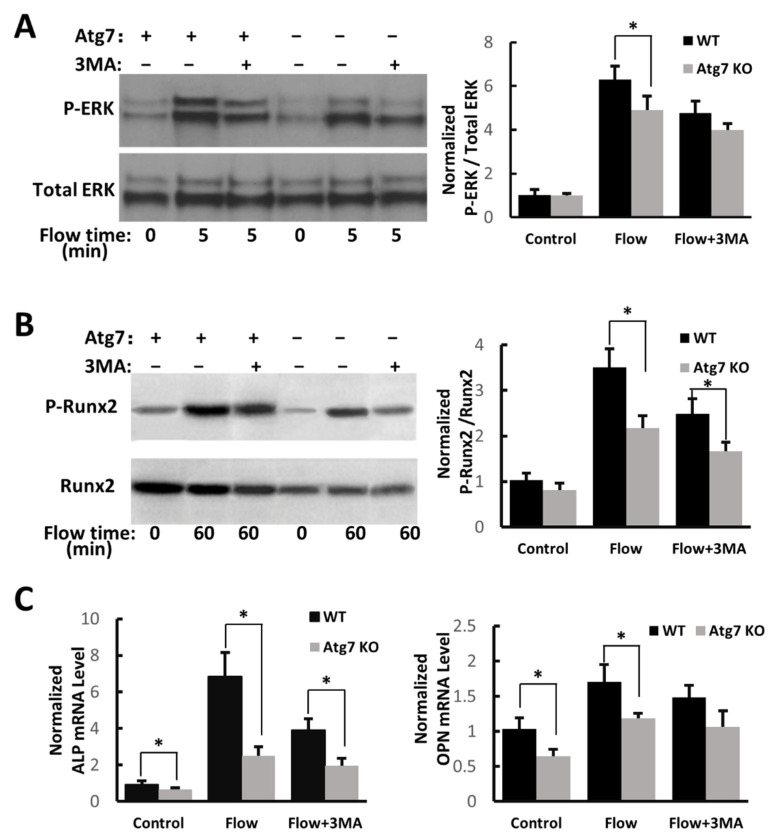
Autophagy deficiency affects mechanical loading-induced osteoblast responses. (**A**) Western blot of ERK1/2 phosphorylation in WT and Atg7 KO osteoblasts with and without 3 MA. (**B**) Western blot of Runx2 phosphorylation in WT and Atg7 KO osteoblasts with and without 3 MA. (**C**) Quantified mRNA levels of ALP, and osteopontin. (*n* > 3, * *p* < 0.05; each bar represents the mean ± SEM).

We found that the level of ERK1/2 phosphorylation in wild-type cells after mechanical loading was significantly higher than that in Atg7 Ko cells. After 3 MA treatment, this ERK1/2 phosphorylation in wild-type cells was significantly reduced (Figure 4A). After 3 MA treatment, Runx2 phosphorylation in wild-type cells was significantly reduced. Additionally, we found that wild-type cells had a higher basal level of Runx2 expression, and Runx2 phosphorylation in wild-type cells was significantly higher than that in KO cells (Figure 4B). We also examined ALP and OPN gene expression in response to mechanical loading, and found that autophagy-deficient osteoblasts have lower expression (Figure 4C). Therefore, our results suggested that autophagy may affect bone formation resulted from mechanical loading.

## 4. Discussion

Autophagy plays an important role in osteoblast function, and impaired autophagy has been implicated in a number of bone diseases [29,30,31]. Previous studies showed that autophagy is involved in bone growth and bone remodeling [8,32], and could ameliorate hindlimb unloading-induced bone loss [33]. In the present study, we showed that autophagy deficiency in mouse bone leads to decreased bone volume, bone ultimate breaking force, and bone stiffness. This bone phenotype is consistent with earlier micro-CT studies of bone structure after autophagy impairment [8,34]. Moreover, bone marrow cells from Atg7 cKO mice had decreased mineralization capacities based on ALP and calcium staining results. This was similar to findings from previous literature, which used UMR-106 and MC3T3-E1 cell lines [10,35]. We also found that autophagy-deficient osteoblasts had reduced Runx2 and osteopontin, suggesting that autophagy may regulate bone growth and remodeling by regulating osteoblast activities. However, there has been a lack of information on the relationship between mechanobiology and autophagy processes within osteoblasts.

To examine the relationship between autophagy and mechanical loading, we further studied the effects of autophagy on osteoblast responses to mechanical loading. With mouse ulna compression tests, we found that the relative bone formation rate purely induced by external loading was significantly higher in wild-type mice compared to Atg7 cKO mice. Together with above in vitro osteoblast differentiation and mineralization data, our results suggest that autophagy may regulate osteoblast metabolism though multiple pathways, with or without the involvement of mechanical loading.

We then examined the role of ATP release in WT and Atg7 KO osteoblasts. We found that autophagy-deficient osteoblasts had reduced amounts of ATP release. This result was similar to earlier findings using MLO-Y4 cells [13]. When adding autophagy inhibitor 3 MA to cells, WT osteoblasts also showed a significant decrease in ATP release, suggesting that autophagy may be involved in this process. We also confirmed that mechanically induced ATP could downregulate P-ERK1/2, Runx2, ALP, and OC expression, which were all shown to be important for osteoblast differentiation and mineralization [22]. Additionally, we found that Atg7-deficient osteoblasts had reduced P-ERK1/2 and P-Runx2 expression, as well as reduced ALP and OPN mRNA expression, in response to mechanical loading. When using 3 MA to inhibit autophagy in cells, WT osteoblasts also had significant decreases in the above protein and gene expressions in response to mechanical loading, highlighting the possible involvement of autophagy in the above responses.

Autophagy and ERK1/2 are closely associated with each other [36,37], and the ATP-ERK1/2 pathway is a major pathway in osteoblast mechanotranduction able to initiate many other osteoblast responses [38]. Therefore, autophagy may be involved in osteoblast responses to mechanical loadings through ATP release. Moreover, there are many cellular components related to both cell autophagy and mechanobiology, such as primary cilia and cytoskeleton. Given that autophagy plays a crucial role in global cell metabolism, it is not surprising if other signaling pathways are also involved in both osteoblast autophagy and mechanotransduction [39,40,41,42,43].

## 5. Conclusions

In summary, autophagy was demonstrated to be involved in bone growth and remodeling through multiple pathways with or without influence from direct mechanical stimulation on osteoblast. Autophagy-deficient mice had a reduced relative bone formation rate induced purely by mechanical loading, indicating possible autophagy involvement in osteoblast mechanotransduction. Additionally, we showed that autophagy affects mechanical loading-induced ATP release, ERK1/2 phosphorylation, Runx2 phosphorylation, and other gene expression, suggesting that the ATP-ERK1/2 pathway may play an important role in the possible involvement of autophagy in osteoblast mechanotransduction.

## Data Availability

Not applicable.
